# Cellular Functions of Deubiquitinating Enzymes in Ovarian Adenocarcinoma

**DOI:** 10.3390/genes14040886

**Published:** 2023-04-09

**Authors:** Yosuk Min, Hong-Beom Park, Kwang-Hyun Baek, Sohyun Hwang

**Affiliations:** 1 Department of Biomedical Science, CHA University, Seongnam 13488, Gyeonggi-do, Republic of Korea; 2 Department of Pathology, CHA Bundang Medical Center, CHA University School of Medicine, Seongnam 13496, Gyeonggi-do, Republic of Korea; 3 CHA Future Medicine Research Institute, CHA Bundang Medical Center, Seongnam 13496, Gyeonggi-do, Republic of Korea

**Keywords:** deubiquitinating enzyme, epithelial ovarian cancer, ubiquitin–proteasome system

## Abstract

In ovarian cancer patients, the 5-year survival rate is 90% for stages I and II, but only 30% for stages III and IV. Unfortunately, as 75% of the patients are diagnosed at stages III and IV, many experience a recurrence. To ameliorate this, it is necessary to develop new biomarkers for early diagnosis and treatment. The ubiquitin–proteasome system is a post-translational modification that plays an important role in regulating protein stability through ubiquitination. In particular, deubiquitinating enzymes (DUBs) regulate protein stability through deubiquitinating substrate proteins. In this review, DUBs and substrates regulated by these enzymes are summarized based on their functions in ovarian cancer cells. This would be useful for the discovery of biomarkers for ovarian cancer and developing new therapeutic candidates.

## 1. Introduction

### 1.1. Ovarian Cancer

Ovarian cancer is the 7th most frequent cancer in women globally [1]. As the rates of ovarian cancer diagnosis rate per 100,000 people per year and the death rate of patients are consistently decreasing, the 5-year related survival rate increased to 54.31% in 2020 from 34.38% in 1975 [1,2]. However, 233,565 newly diagnosed patients are in U.S [1]. The 5-year survival rate for stages I and II ovarian cancers, according to the International Federation of Gynecology and Obstetrics (FIGO) staging system, is between 80 and 90%, but that for stages III and IV is only 30% [1,3,4]. This is because stage I and II ovarian cancers localize near the ovaries or fallopian tubes, while stages III and IV cancers invade nearby organs, such as the lymph nodes, abdomen, omentum, liver, spleen, and lung [5,6]. Moreover, 75% of ovarian cancer patients are diagnosed in advanced stages (stage III and IV), due to a deficiency of screening for early prognosis and the absence of precise pelvic or abdominal symptoms [7,8].

#### 1.1.1. Subtypes

Ovarian cancer is subdivided into epithelial and non-epithelial cancer. Epithelial ovarian cancer (EOC) accounts for over 90% of ovarian cancer and has four histological subtypes: serous, endometrioid, clear cell, and mucinous [1,9]. EOC has two categories: type I and type II. Type I EOC has mild symptoms, grows slowly, has a clear borderline, is diagnosed in the early stage, and includes low-grade serous, low-grade endometrioid, mucinous, and clear cell carcinomas. Type II EOC is highly lethal, invasive, grows rapidly, and contains high-grade serous and high-grade endometrioid carcinomas (Table 1) [9,10,11].

#### 1.1.2. Risk Factors

Ovarian cancer involves various risk factors, such as gene mutations, ovulation, dietary factors, and ethnical groups [1]. Some factors increase the risk, while others decrease it. For example, early menarche and late menopause are associated with an increased risk of ovarian cancer, because they cause an inflammatory reaction through the course of many ovulations [27,28]. Moreover, germline mutations in *BRCA1* or *BRCA2* increase the incidence of ovarian cancer. Older age and endometriosis are also risk factors for ovarian cancer [29]. Endometriosis is related to endometriosis-associated EOC [30]. In contrast, early menopause, late menarche, hysterectomy, high levels of vitamin D, and the use of oral contraceptives lead to a decreased incidence of ovarian cancer [1,8,31].

#### 1.1.3. Diagnosis and Screening

Three methods are used for ovarian cancer screening: (i) monitoring biomarkers, (ii) scanning with radiological methods, and (iii) performing cancer tissue biopsy. Cancer antigen 125 (carbohydrate antigen, mucin-16, CA125) is one of the most frequently used biomarkers for ovarian cancer. However, CA125 is inadequate for the early diagnosis of ovarian cancer because of its low sensitivity [32]. To improve the sensitivity of screening ovarian cancers, new biomarkers such as human epididymis protein 4 (HE4), are identified [33]. Monitoring the expression of HE4 is a more sensitive screening method than monitoring CA125, but it is still inadequate [34]. For these reasons, screening algorithms, such as the risk of malignancy index (RMI) and risk of ovarian malignancy algorithms (ROMA), have been developed [34,35]. 

Among radiological scans, transvaginal sonography (TVS) is more sensitive to detecting ovarian cancer than computed axial tomography (CT) or other radiological methods [36,37]. Finally, ovarian cancer is definitively diagnosed by biopsy during surgery or core-needle biopsy [1]. However, the core–needle biopsy is not recommended because of the risk of metastasis [38]. 

#### 1.1.4. Treatments

Standard treatments for ovarian cancer are chemotherapy and surgery. Currently, there are two types of chemotherapy (platinum-containing drugs and taxane) and two types of targeted treatments which are poly ADP-ribose polymerase (PARP) inhibitors (PARPi) and vascular endothelial growth factor (VEGF) inhibitors (VEGFi) for ovarian cancer, depending on their anti-tumor mechanism or material [1]. First, platinum-containing drugs (including cisplatin, carboplatin, oxaliplatin, picoplatin, and satraplatin) cause DNA damage and induce apoptotic cell death by binding to the N7 of guanine bases, resulting in intrastrand adduction and interstrand crosslinks [11,39]. Cisplatin was first used in 1971 and was the first platinum-based drug approved by the Food and Drug Administration (FDA) in 1978 [40]. However, patients resistant to platinum therapy have lower median survival rates. The median survival of platinum-sensitive patients is two years, but that of platinum-resistant ovarian cancer is 9–12 months [41]. Second, taxanes, including paclitaxel and docetaxel, target microtubules to inhibit cell division [42,43]. Unlike other microtubule-targeted drugs, taxanes stabilize microtubules and repress the microtubule dynamics [44]. In 1992, the FDA approved taxol for the treatment of ovarian cancer [45]. 

As targeted treatments, PARPi has been suggested as the mechanism of synthetic lethality that *BRCA*-deficient tumor cells become sensitive to PARPi [46]. In 2014, olaparib was approved by the FDA for the treatment of ovarian cancer. Talazoparib, niraparib, and rucaparib have also been approved by the FDA as PARPis [47,48]. Moreover, angiogenesis-targeted drugs inhibit angiogenesis by inhibiting VEGF, mainly using monoclonal VEFG antibodies, bevacizumab, and VEGF receptor tyrosine kinase inhibitor (VEGF RTKi) [9]. The FDA approved bevacizumab for the treatment of advanced-stage ovarian cancer in 2018 [49]. Additionally, VEGF RTKi, cediranib, and nintedanib have been studied for use in patients with ovarian cancer [50,51].

Ovarian cancer treatment depends on several conditions, such as stage, *BRCA* mutation, and platinum sensitivity of the patients [1,9]. A combination of appropriate drugs was selected for each patient, and intraperitoneal chemotherapy and neoadjuvant chemotherapy were also considered [52].

The surgery for the treatment of ovarian cancer patients varies according to the stage of ovarian cancer [5]. Total abdominal hysterectomy and salpingo-oophorectomy are the most commonly used methods; however, surgery such as infracolic omentectomy, selective lymphadenectomy, and cytoreductive surgery can be performed [5,53]. 

### 1.2. Ubiquitin, Ubiquitin–Proteasome System (UPS), and Deubiquitinating Enzymes (DUBs)

**Figure 1:**
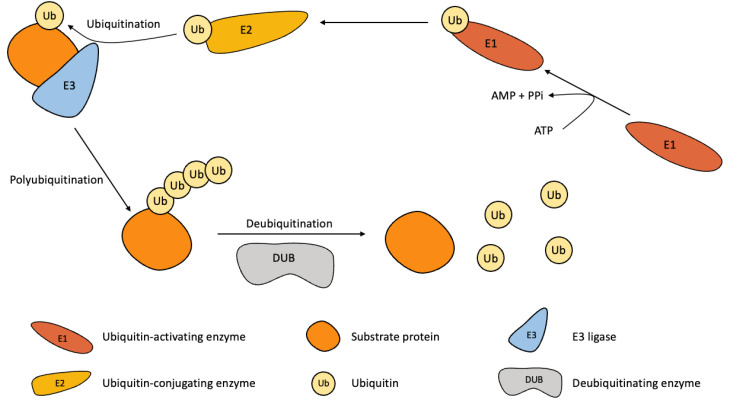
A mechanism of the ubiquitination and deubiquitination. The substrate is ubiquitinated by the E1 ubiquitin-activating enzyme, E2 ubiquitin-conjugating enzyme, and E3 ligase. The polyubiquitin chain is formed by successive mechanisms. A substrate with polyubiquitin chains regulates various cellular functions. Conversely, the polyubiquitin chain of the substrate is cleaved by the DUB.

Ubiquitin, composed of 76 amino acids, binds with substrate and forms chains by forming covalent bonds in Lys and Met residues [54,55,56]. Attaching ubiquitin itself is a mechanism known as polyubiquitination. Polyubiquitination is associated with seven lysine residues (Lys6, Lys11, Lys 27, Lys29, Lys33, Lys48, and Lys63) and one methionine residue (Met1) [57,58]. Ubiquitination is mediated by three enzymes: E1 ubiquitin-activating enzyme, E2 ubiquitin-conjugating enzyme, and E3 ubiquitin ligase. Firstly, E1 binds to ubiquitin using ATP, and through the E2, E3 ligase attaches ubiquitin to its substrate protein (Figure 1) [57,59,60]. 

#### 1.2.1. Ubiquitination

Ubiquitination or polyubiquitination is a post-translational modification (PTM). The subtypes of ubiquitin modifications are monoubiquitination, multi-monoubiquitination, polyubiquitination, mixed-polyubiquitination, and branched polyubiquitination [61,62]. Ubiquitination affects many cellular functions, such as protein degradation, DNA damage response (DDR), cell cycle control, apoptosis, autophagy, endocytosis, and cellular signaling [62,63,64,65,66], and each ubiquitin residue plays different roles in cells. For instance, Met1-linked ubiquitination regulates NF-κB signaling pathway, anti-apoptosis, and hypoxic stress; Lys6-linked polyubiquitin is associated with mitophagy; Lys11-linked ubiquitin chain regulates cell division; Lys27-linked polyubiquitination blocks the activation of the inflammasome; Lys29-linked diubiquitin regulates the cell cycle and proteotoxic stress; Lys33-linked polyubiquitination is associated with DNA methylation; Lys48-, branched Lys63 and Lys48-linked polyubiquitin chains are related to proteasomal degradation; and Lys63-linked polyubiquitination regulates DNA damage repair and PI3K/Akt signaling [67,68,69,70,71,72,73,74,75,76].

#### 1.2.2. UPS

UPS is composed of ubiquitin E1, E2, E3, and 26S proteasomes. Through this mechanism, substrate proteins, which have a polyubiquitin chain, are degraded through the 26S proteasome using ATPs [77,78]. The proteasome is composed of the 20S proteasome, which is the core of proteolysis, and various proteasome regulators (e.g., PA700, PA28αβ, PA28γ, P200, ECM29, and PSMF1) [79]. UPS dysfunction occurs in many diseases, such as neurodegenerative diseases, autoimmune diseases, diabetes, and cancer [80,81]. Thus, UPS components are a target of diseases, specifically cancers that induce apoptosis and cell cycle arrest [82,83].

#### 1.2.3. DUBs

DUBs detach ubiquitin from substrate proteins, and proteins that lose the polyubiquitin chain (Figure 1) [84,85]. DUBs are regulated by PTM such as phosphorylation, ubiquitination, SUMOylation, lipid modification, and O-glycosylation. These PTMs regulate the stability, interaction affinity, localization, and catalytic activity of DUBs [86]. There are nine subfamilies divided by structural fold, the process of action, and sequence motifs [87]. These nine subfamilies are subdivided into eight Cys proteases and one metalloprotease, where protease targets proteolysis [88]. The eight Cys protease subfamilies are ubiquitin-specific proteases (USP), ovarian tumor proteases (OTU), ubiquitin C-terminal hydrolases (UCH), Machado-Josephin domain proteases (MJD), monocyte chemotactic protein-induced protease (MCPIP), permuted papin fold-peptidase of dsDNA and eukaryotes (PPPDE), motifs interacting with Ub-containing novel DUB family (MINDY), and zinc finger with UFM1-specific peptidase domain protein (ZUFSP). One metalloprotease subfamily is the JAB1/MPN/MOV34 metalloenzymes (JAMM) [87,89,90,91].

#### 1.2.4. UPS and DUBs in Cancer

DUBs and the UPS are related to several diseases, such as neurodegeneration, developmental disorders, and cancers [65]. Specifically, ubiquitination of oncoproteins and deubiquitination of tumor suppressor proteins may lead to tumorigenesis suppression, tumor progression, cancer metabolism, and chemotherapy or radiotherapy resistance, whereas deubiquitination of oncoproteins causes cancer by prolonging the half-life of oncoproteins [62,89,92]. For instance, deubiquitination of FOXM1 induces cancer progression and chemoresistance through the DUB UCHL3; OTUD1 reduces the pro-oncogenic TGF-β signaling mediated by SMAD7 to suppress the metastasis in breast cancer [93,94].

## 2. DUBs in Ovarian Cancer

Fifteen different DUBs and their 17 substrates have been investigated in ovarian cancer (Table 2). Most DUBs have been studied in high-grade serous and endometrioid carcinomas, while non-epithelial, mucinous, and clear cell carcinomas have rarely been studied. Surprisingly, all DUBs related to ovarian cancer were overexpressed and seemed to act like an oncoprotein. Therefore, mechanistic studies of these DUBs, their substrates, and intracellular functions could be the basis for EOC treatment.

### 2.1. DUBs Associated with Cell Cycle Arrest

The cell cycle in cancer cells is unscheduled because of the scarcity of checkpoints, owing to chromosomal and genomic instability [114]. Cell cycle arrest and control by checkpoints are important for cancer growth control. Cell cycle checkpoints are regulated by various cyclin-dependent kinase (CDK) and cyclin families. The G1/S checkpoint is activated by certain stimuli (such as hormones, ultraviolet (UV), replicative senescence, and growth factors) which inhibit the CDK4/CDK6/Cyclin D complex or CDK2/Cyclin E complex through p15, p16, p18, p19, and p27 [114,115,116,117]. The G2/M checkpoint is activated by DNA damage such as UV radiation, platinum-based therapy, or infrared radiation (IR) [118]. CDC2/Cyclin B complex promotes the cell cycle from the G2 phase to the M phase. This complex is inhibited by p53 via p21 and GADD45 [119,120]. However, when DNA damage could not be repaired appropriately, programmed cell death would be induced by checkpoint signaling [115].

**Figure 2:**
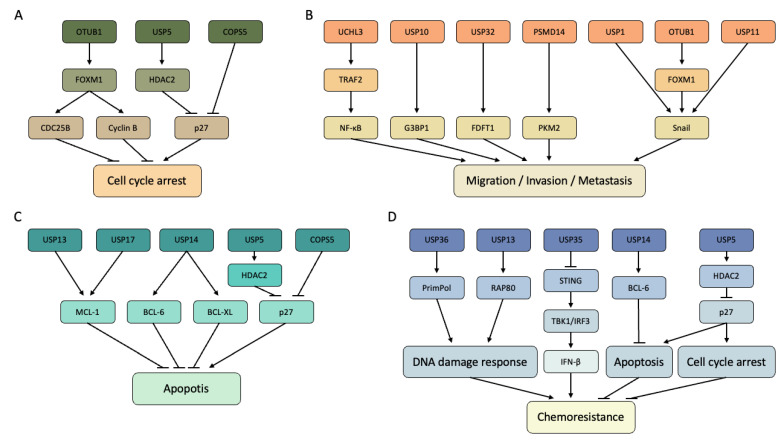
DUBs, substrates, and their functions. (**A**) DUBs and substrates associated with cell cycle arrest. (**B**) DUBs and substrates associated with migration, invasion, and metastasis. (**C**) DUBs and substrates associated with apoptosis. (**D**) DUBs and substrates associated with DNA damage response and chemoresistance. The arrows from the DUBs to the substrates indicate that the binding between them is confirmed, and the stability of the substrate is controlled by DUBs.

COPS5 and USP5 negatively regulate the stability of p27 (Figure 2A) [95,104,105]. p27 induces cell cycle arrest by inhibiting the CDK2/Cyclin E complex at the G1/S checkpoint [119]. Moreover, USP5 positively regulates the stability and deubiquitinates HDAC2, of which the mutation or loss of function acts as an oncogene (Figure 2A) [95,121]. OTUB1 positively regulated CDC25B and Cyclin B via the control of FOXM1 (Figure 2A) [109]. FOXM1 promotes the transcription of both genes to their target promoters [122,123]. Moreover, FOXM1 promotes the E2F pathway by regulating *CDC25B* gene transcription [122]. Between CDC25B and Cyclin B, CDC25B dephosphorylates CDK1/Cyclin B to induce the cell cycle from G2 to M [124,125].

### 2.2. DUBs Associated with Migration, Invasion, and Metastasis

Metastasis in cancer is a critical event that affects the survival of patients and is one of the standards of cancer progression. In EOC, there are three ways for metastasis in ovarian cancer: transcoelomic, hematogenous, and lymphatic routes, of which hematogenic, motility, and lethal routes are the most common [126]. Metastasis occurs through epithelial–mesenchymal transition (EMT) in cancer cells through the exchange of cadherin and integrin expression levels [127]. To investigate cancer metastasis in vitro, migration assay and invasion assay were performed, and the EMT-associated markers, such as E-cadherin, N-cadherin, ZEB, Snail, and Twist were verified [128,129].

USP1, OUTB1, and USP11 induced migration, invasion, and metastasis through Snail (Figure 2B) [109,111,112]. OTUB1 positively regulates FOXM1, a transcription factor that promotes *Snail* transcription. FOXM1 regulates EMT, cell migration, and drug resistance [130]. Snail is also a transcription factor that promotes EMT and motility of cells [131]. Snail regulates the expression of E-cadherin, N-cadherin, fibronectin, and vimentin to induce EMT [132]. As Snail is usually overexpressed in cancer tissue and especially metastatic tissue, patients with the highly expressed Snail exhibited the short survival expectancy [133,134].

TRAF2 was deubiquitinated and positively regulated by UCHL3 (Figure 2B) [110]. NF-κB which is regulated by TRAF2, belongs to a family of five master transcription factors. Twist, adhesion molecules, and other EMT-associated molecules are the targets of NF-κB [135]. USP10 regulates GTPase-activating protein SH3 domain-binding protein 1 (G3BP1) (Figure 2B) [102]. G3BP1 is an RNA-binding protein that affects the stability of mRNA in response to extracellular stimuli. G3BP1 responds to cellular stress and activates the STING pathway by binding to dsDNA and RNA in the cytosol [136]. In addition to inducing metastasis in EOC, G3BP1 induces metastasis through IL-6/G3BP1/STAT3 signaling in renal cell carcinoma [137]. FDFT1 stability is regulated by USP32, and this mechanism is associated with mesenchymal markers, such as FN1, ZEB1, Snail, and CDH2 (Figure 2B) [103]. In various cancers, including ovarian cancer, FDFT1 is associated with invasion and activates cancer cell metastasis through MMP1, CD44, and AKT/mTOR/HIF1α signaling [138]. PSMD14 deubiquitinated PKM2 and positively regulated the PKM2 monomer (Figure 2B) [106]. PKM2 promotes EMT by inhibiting E-cadherin expression and cancer metastasis along with PAK2 and HSP90 [139].

### 2.3. DUBs Associated with Apoptosis

Apoptosis is a caspase-dependent programmed cell death promoted by reactive oxygen species (ROS), UV radiation, chemotherapy, and other stimuli [140,141]. In ovarian cancer, patients are treated by radio and chemotherapy, which induce apoptosis. Specifically, radiotherapy and platinum-based therapies induce DNA damage [41]. DNA damage, one of the causes of the intrinsic apoptotic pathway, promotes p53 overexpression [39]. Overexpression of p53 activates the p53-dependent apoptotic pathway, which activates BAX, BAK, mitochondrial outer membrane permeabilization (MOMP), apoptotic protease activating factor 1 (APAF1), caspase-9 to form an apoptosome and cause apoptosis [141,142]. Moreover, taxane causes cell cycle arrest by blocking the cell division and promotes apoptosis through the abnormal activity of c-Jun N-terminal kinase/stress-activated protein kinase (JNK/SAPK), BCL-2, and p53 pathway [143,144].

In ovarian cancer, USP13, USP17, and USP14 positively regulate the stability of MCL-1 and BCL-XL, which are members of the anti-apoptotic BCL-2 family (Figure 2C) [96,100,107]. As MCL-1 and BCL-XL suppress BAX and BAK to inhibit the intrinsic apoptosis pathway, the expression levels of USP13, USP17, and USP14 are important. Therefore, it is predicted that upregulation of MCL-1 and BCL-XL would result in poor treatment because of inhibiting apoptosis in cancer cells [145]. USP14 also positively regulates the stability of BCL-6, a proto-oncogene that inhibits the expression of p53; consequently, p53-dependent apoptosis is suppressed (Figure 2C) [97,146,147]. USP5 negatively regulated the stability of p27 by modulating the HDCA2 expression (Figure 2C) [95]. HDAC2 and COPS5 downregulate p27, a regulator of CDK/Cyclin, which is associated with apoptosis via cell cycle arrest [148].

### 2.4. DUBs Associated with DNA Damage and Chemoresistance 

DDR occurs when cells experience DNA dagame through stimuli, and the repair mechanism of DDR varies depending on which damage occurs. There are five main DDR mechanisms: homologous recombination (HR), base excision repair (BER), mismatch repair (MMR), nucleotide excision repair (NER), and nonhomologous end joining (NHEJ) [149]. In the course of ovarian cancer treatment, cisplatin leads to the induction of BER, NER, HR, and NHEJ through the DNA adduction, DNA crosslinking, and the production of reactive oxygen species [150,151,152,153,154,155]. 

USP13 regulates and deubiquitinates RAP80, which regulates HR through BRCA1, BRCC46, and Abraxas (Figure 2D) [101,156]. USP36 deubiquitinates and regulates the stability of PrimPol, a DNA damage-tolerant polymerase (Figure 2D) [108]. PrimPol synthesizes the DNA primers to restart and reprime DNA replication during replication stress [108,157]. PrimPol is also involved in DDR as the component of the Fanconi anemia pathway to repair DNA by intra-strand crosslink [158]. 

Chemoresistance could be caused by a combination of several complex factors. In this review, we focused on the combination of DUBs and their substrates that leads to chemoresistance (Figure 2D). USP14 inhibits apoptosis by deubiquitinates BCL-6. USP5 inhibits cell cycle arrest by deubiquitinates HDAC2. PrimPol which is deubiquitinated by USP36 increases DNA replication. RAP80 deubiquitinated by USP13 promotes DDR through BRCA1. These studies have also shown that chemoresistance could be induced by overexpression of these DUBs and their substrates [95,97,101,108]. Lastly, USP35 suppressed anti-tumor immunity of type I interferon through the STING/TBK1/IRF3 pathway and as a result, chemoresistance was induced [113,159,160,161]. 

### 2.5. Other DUBs

Mutated *p53* promotes EMT, receptor tyrosine kinase signaling, and interactions with the extracellular matrix [162]. In particular, the p53 (R175H) cancer cell growth through the GEF-H1 pathway and cell cycle in EOC [163,164]. Mutated p53 (R157H) is deubiquitinated by USP15 [98]. The half-life of deubiquitinated p53 (R175H) increases and aggregates to show a gain of functions. 

USP13 regulates the stability of oxoglutarate dehydrogenase (ODGH) and ATP citrate lyase (ACLY) through deubiquitination [99]. ODGH mediates the conversion of decarbonized α-ketoglutaric acid to fumarate and ACLY lysis of citrate to acetyl-CoA. *USP13* knockdown inhibits both mechanisms, resulting in mitochondrial and lipogenic dysfunctions. Therefore, USP13 suppression decreases ATP production by repressing the TCA cycle and inhibiting *de novo* lipogenesis [99].

## 3. Discussions

Early diagnosis of ovarian cancer is critical for patient survival. The survival rates of patients with stage I and II cancer were high, but the survival rates of patients with stage III and IV cancer were low. However, most patients were diagnosed with stages III and IV [165]. Therefore, diagnosis through serum tests, such as CA125 and HE4, is important. TVS often needs to be performed for accurate examination; however, because it has some limitations, it is quicker and more convenient to detect cancers during a blood test. The diagnostic rate of serum tests based on CA125, HE4, and ROMA is quite high, but it is still necessary to develop biomarkers that could reliably detect cancer in serum [166]. Because DUBs showed elevated expression in several cancers, they may be used as biomarkers. Mainly USP4, USP7, USP22, UCHL1, UCHL5, and OTUB1 could be used as biomarkers for each type of cancer such as EOC, breast cancer, melanoma, cervical cancer, and colorectal cancer [167]. The DUBs in Table 2 could be used as biomarkers for ovarian cancer; however, serum detection is required. This is because core-transvaginal needle biopsy is not commonly used because of the risk of metastasis [38]. In addition, identifying biomarkers to predict diagnosis or targeted therapy through postoperative patient sample analysis is also very important for improving patient survival.

All DUBs listed in this review (Table 2) are elevated in cancer; therefore, DUB inhibitors could be used to treat patients. Many DUB inhibitors, such as P5091, inhibits USP7, Sputin1, which inhibits USP10 and USP13, and LDN-57444, which inhibits UCHL1 and UCHL3 [167]. In addition, the DUB inhibitor VLX1570, which inhibits USP14 and UCHL5, has undergone phase 1 clinical trials in multiple melanoma patients [168]. However, because the characteristic of DUB inhibitors is that one DUB should regulate multiple substrates, side effects could be severe when drugs targeting DUB are treated. To overcome this limitation, DUB-tailored inhibitor synthesis based on ultra-high throughput screening technology has been suggested as an innovative method for finding selective DUB inhibitors [169].

As well as finding selective DUB inhibitors, research has focused on the development of proteolysis-targeting chimera (PROTAC) and deubiquitinase-targeting chimera (DUBTAC), to reduce their side effects. PROTAC or DUBTAC is composed of a ligand of E3 ligase or DUB, a linker, and a ligand of the substrate. These molecules promote the binding and activity of E3 ligases or DUBs to substrates [170,171]. One of the PROTAC, ARV-110, which targets androgen receptors in prostate cancer, completed phase 2 clinical trials, and ARV-471, which targets estrogen receptor in breast cancer, also completed phase 2 clinical trials [172]. Based on this notion, it could be used as an adjuvant drug by reducing the half-life of the targeted oncoprotein or increasing the stability of the anti-cancer protein. In addition, it might be possible to make cells chemosensitive by targeting proteins related to chemoresistance. Therefore, it would be effective to develop PROTAC and DUBTAC as well as DUB inhibitors because they could reduce side effects and target multiple proteins with similar functions by targeting a specific domain. 

This review has several limitations. Firstly, we summarized only DUBs with their known substrates in ovarian cancers. There are more studies on identifying important DUBs such as USP8 and USP19 in ovarian cancers [173,174]. However, in this paper, we could not include them, as their target substrates and underlying mechanism were unknown. In addition, we could not include well-known mechanisms of DUBs in other cancers. For example, USP1 deubiquitinates FANCD2/FANCI or PCNA to prevent the recruitment of interstrand crosslink repair proteins and translesion DNA polymerase to inhibit DNA repair; USP7 regulates lung squamous cell carcinoma cell proliferation through MEK/ERK signaling by deubiquitinates the Raf-1 [175,176]. These well-known DUBs could also have key roles in ovarian cancers through these mechanisms, but it further needs to be confirmed experimentally. 

## 4. Conclusions

In this review, we have organized 15 DUBs, 17 substrates, and their functions in EOC. This could support the development of biomarkers or the search for therapeutic targets. The functions of DUBs via regulating substrates, which are oncoproteins or anti-cancer proteins, are indispensable for revealing the mechanisms in ovarian cancer cells. Among the 100 DUBs that were identified, only 15 have been investigated in EOC. Therefore, further research is needed to overcome EOC.

## Figures and Tables

**Table 1:** Subtypes of ovarian cancer and characteristics of each.

Cancer	Type	Subtype	Grade	Characteristics	References
Ovarian cancer	Epithelial	Serous	High-grade	90% of serous ovarian cancer	[10,12,13,14,15]
				Generally diagnosed in older women	
				Present at advanced stages	
				Have poor prognosis (10-year survival rate of 30%)	
				Originates in the ovary, fallopian tube, and others	
				Have *TP53* and *BRCA* mutation	
				50% of tumors have homologous recombination deficiencies	
			Low-grade	Usually diagnosed in younger women	[10,12,14,16,17]
				Grow slowly	
				Have a better prognosis than high-grade serous carcinoma	
				Originates in ovary	
		Endometrioid	Low-grade	Chemosensitive	[10,18,19]
				Generally diagnosed in the early stage	
				Originates in endometriosis	
				Has better prognosis	
		Mucinous	Low-grade	Most uncommon cancer in epithelial ovarian cancer	[10,19,20,21]
				Associated with metastasis from the gastrointestinal tract	
				Most patients are diagnosed with stage I	
		Clear cell	Low-grade	Relatively have a good prognosis	[1,10,19,22]
				Generally diagnosed in the early stage	
				Have resistance to platinum-based chemotherapy in advanced stage	
	Non-epithelial	Germ cell	-	3% of ovarian cancer	[1,10,23,24]
				Have obvious makers of tumor	
				Diagnosed at a young age (10~30 years old)	
				Histologically like men’s germ cell tumors in the testes	
		Sex cord-stromal	-	Under 2% of ovarian cancer	[25,26]
				Generally diagnosed in the early stage	
				Smoking decreases the risk of the tumor	
				Rarely have malignance	

**Table 2:** List of DUBs, substrates, and functions which are associated with ovarian cancer.

DUBs	Subtypes of Cancer	Substrates	Functions	Proteins Related to Function	Expression in Cancer	Level of Expression	References
USP5	Serous, endometrioid	HDAC2	Cell cycle arrest, chemoresistance, apoptosis	p21, p27	Up	mRNA, protein	[95]
USP14	Serous, endometrioid	BCL-XL	Apoptosis, cell cycle arrest	PCNA, Cyclin A, Cyclin D1	Up	Protein	[96]
	Serous, endometrioid	BCL-6	Chemoresistance apoptosis,	Not determined	Up	Protein	[97]
USP15	Serous, endometrioid	p53 (R175H)	Tumor cell death	Not determined	Up	mRNA	[98]
USP13	Serous	ACLY, OGDH	Glutaminolysis, mitochondrial dysfunction, lipid synthesis	Not determined	Up	Copy number	[99]
	Serous, clear cell	MCL-1	Apoptosis	Not determined	Up	Protein	[100]
	Serous, mucinous	RAP80	DNA damage response, chemoresistance	BRCA1, CCDC98	Up	mRNA, protein	[101]
USP10	Serous, endometrioid	G3BP1	Cell proliferation, migration, invasion	Not determined	Up	mRNA	[102]
USP32	Serous, endometrioid	FDFT1	Tumor sphere formation (invasion)	ZEB1, FN1, CDH1, CDH2, Snail 1	Up	Protein	[103]
COPS5	Serous, endometrioid	p27	Cell proliferation, apoptosis	Akt	Up	mRNA, protein	[104,105]
PSMD14	Serous, endometrioid	PKM2	Cell viability, migration, invasion	Not determined	Up	mRNA, protein	[106]
USP17	Serous, clear cell, endometrioid	MCL-1	Chemoresistance, apoptosis	MGMT	Up	Protein	[107]
USP36	Serous, endometrioid	PrimPol	Chemoresistance, DNA replication stress	Not determined	Up	Protein	[108]
OTUB1	Serous, endometrioid	FOXM1	Cell cycle arrest, invasion	Snail, CDC25B, Cyclin B	Up	Protein	[109]
UCHL3	Serous, endometrioid	TRAF2	Inflammatory response, tumorigenesis, migration	NF-κB1	Up	mRNA, protein	[110]
USP11	Serous	Snail	Cell proliferation, migration, invasion,	E-cadherin, N-cadherin, Fibronectin	Up	mRNA, protein	[111]
USP1	Serous, clear cell, endometrioid	Snail	Invasion, metastasis	ATM, ATR	Up	Protein	[112]
USP35	Serous	STING	Chemoresistance	TBK, IRF3, IFN-β	Up	Protein	[113]

## Data Availability

No new data were created in this study. Data sharing is not applicable to this article.
